# Supplementary material for: Breathing–Swallowing discoordination after definitive chemoradiotherapy for head and neck cancers is associated with aspiration pneumonia
Source: PLoS One. 2024 Jul 11;19(7):e0305560. doi: 10.1371/journal.pone.0305560 (PMC11238977; doi:10.1371/journal.pone.0305560)
Supplement: Attachment — Submitted filename: Response to Reviewers.docx [file pone.0305560.s002.docx]

**Dear Editors and Reviewers**

We sincerely appreciate the editor’s and reviewers’ comments. We revised our article according to the comments.

Reviewer #1:

The authors provided an interesting observational single center study on breathing-swallowing discoordination after definitive chemoradiotherapy for head and neck cancers. The manuscript provides original data on a novel topic. However, there are some issues that need to be addressed with a revision:

Response: Thank you for your thoughtful review.

Comment:

1. The introduction section is quite brief. Please provide additional background in order to justify the rationale and aims of the study.

Response: Thank you for your valuable opinion. The background was too short, so I have added citations and added more details.

2. Please provide the date of ethical committee approval.

Response: According to this comment, I added 2016-12-12.

3. Please specify the institute were the study was conducted in the methods section.

Response: According to this comment, I have written that the procedure was carried out at Tohoku University Hospital.

4. Plase use the same tamplate for all the tables.

Response: Thank you for your valuable comments. The table templates have been unified.

5. Table 3 should be revised as for layout.
6. Table 4 should be unified as being a single table might increase readability.

Response: I would like to thank you for your very valuable comments. Based on Reviewer 2's comments, we combined Tables 3 and 4 into one and adjusted the layout.

7. Please remove table 5 and provide its content in the text.

Response: According to this comment, Table 5 has been deleted and its contents have been written in lines 231 to 234 of the main text as shown below.

“Among the 21 patients under consideration, 20 exhibited the SW-E pattern before the treatment, whereas only one exhibited the SW-I pattern. After the CRT, 10 individuals manifested the SW-E pattern, and 11 displayed the SW-I pattern. A pronounced disparity was observed after the treatment, with a notable increase in the prevalence of the SW-I pattern (p = 0.00139)”

8.　　The observational design of the study must be included among the limitations of the study.

Thank you for your valuable comments. We wrote it in lines 296 to 302 of the main text as shown below.

“A limitation of this study is its short evaluation period. Thus, a long-term investigation of respiratory dysphagia and swallowing function after CRT is required. Further extensive studies are required to determine whether breathing and swallowing problems after CRT increase the risk of aspiration pneumonia. Individual characteristics, including sex, age, primary site, and stage classification, exhibit variations, introducing potential limitations, such as selection and information biases. Finally, the study’s statistical power and capacity to discern significant effects may have been compromised due to the relatively small sample size of 21 participants.“

Reviewer #2:

Response: Thank you for your thoughtful review.

Comment:

Introduction
1. Provide more background on the prevalence of dysphagia and aspiration pneumonia following chemoradiotherapy (CRT) for head and neck cancers. Cite key statistics on incidence rates. doi: 10.1002/hed.20279 and doi:10.1016/j.jvoice.2021.09.040

Response: According to this comment, We have cited the papers you have presented (citation numbers 2 and 7) and have included the morbidity rate and basic information in the background n lines 52 to 55 of the main text.

2. Explain the pathophysiology leading to swallowing dysfunction after CRT - fibrosis, muscle weakness, sensory changes etc.
Response: According to this comment, we have provided detailed information on lines 65-73 of the main text, including cited references as shown below.

“Typical side effects of CRT include mucositis, pain, xerostomia, edema, long-term muscle atrophy, fibrosis, and sensory loss, all of which decrease a patient’s quality of life [9-14]. In addition to these changes, radiation-induced fibrosis of the pharyngeal mucosa occurs, resulting in decreased tongue strength, reduced tongue base retraction, delayed laryngeal vestibular closure, or problems with swallowing coordination movements, which are some of the most prominent symptoms [15-22]. Consequently, the risk of aspiration and aspiration pneumonia (defined as pneumonia secondary to the inhalation of food particles, saliva, or other foreign objects) increases. The problems associated with swallowing dysfunction are further compounded by the increased risk of aspiration pneumonia due to dysphagia [18, 23].”

3. Discuss the importance of breathing-swallowing coordination in airway protection and how disorders can increase aspiration risk.

Response: According to this comment, we have provided detailed information on lines 78-86 of the main text, including cited references as shown below.

“The coordination of breathing and swallowing is a physiological defense mechanism that prevents aspiration and aspiration pneumonia. Swallowing usually occurs during expiration and subsequent breathing resumes with expiration, and this expiratory-swallow-expiratory (E-SW-E) pattern prevents entry of pharyngeal contents into the lower respiratory tract [26, 27]. The expiratory flow surrounding swallowing prevents the entry of pharyngeal contents into the lower airways. The expiratory flow also facilitates mechanical functions favorable to swallowing, such as elevation and closure of the larynx, generation of pharyngeal pressure with resultant food mass clearance, and opening of the pharyngo-esophageal segment [28]. Conversely, increased incongruence between breathing and swallowing, detected as I-SW and SW-I patterns, is a major risk factor for aspiration pneumonia in patients [26, 27, 29].”

4. Introduce innovative techniques in H&N management like videofluoroscopy, endoscopy for swallow evaluation and exoscope to correct identificate digestive tract for correct reconstruction. Discuss and cite doi:10.3390/jcm11133639 and doi: 10.1007/s00455-022-10484-8.

Response: According to this comment, we have quoted the papers you suggested (cited references 30 and 31) and have written them as follows in Lines 86 to 91 in the main text as shown below.
“Technological advances over the past decade have led to the development of a variety of new devices in the head and neck region, including the EXOSCOPE, to achieve a less invasive, more accurate, and safer approach for preserving function after treatment [30]. However, the gold standards for assessing swallowing function remain videoendoscopy (VE) and videofluoroscopy (VF) [31, 32], and the methods described above for assessing breathing and swallowing coordination are impractical.”

5. Discuss prior research that has analyzed breathing-swallowing patterns in patients with dysphagia using noninvasive monitoring methods.

Response: Thank you very much for your very valuable opinion. Important information was missing. According to this comment, we have written the following on lines 91-99.

“Recently, Yagi et al. developed a swallowing monitoring system as a non-invasive method to examine swallowing-respiration coordination [33]. This system has made it possible to systematically evaluate swallowing sounds, laryngeal movements during swallowing, and coordination of swallowing and breathing at the bedside. Subsequent studies using this system revealed that patients with dysphagia tend to have prolonged swallowing latency and pause duration, and exhibit I-SW or SW-I patterns that reflect dyscoordination of breathing and swallowing [29]. Furthermore, previous studies have shown that breathing-swallowing discoordination is a strong independent predictor of exacerbations in patients with chronic obstructive pulmonary disease (COPD), suggesting that breathing-swallowing discoordination warrants early detection and intervention [34].”

Methods

6. Explain swallowing evaluation methods like Food Intake Level Scale, Hyodo score, Penetration-Aspiration Scale in more detail.
Response: There was insufficient explanation for each item. According to this comment, we have added details.

7. Provide more details on the swallow monitoring system - sensors used, data acquisition, analysis software etc.
Response: Thank you very much for your very valuable opinion. There was insufficient explanation for each item. According to this comment, we have added details on lines 137-148 as shown below.

”Three signal components were recorded by a swallowing monitoring system to detect and evaluate swallowing activity [33]. Figure 1 shows the analytical instrument that was used, and its attachment. Laryngeal motion and swallowing sounds were simultaneously recorded using a custom-made piezoelectric sensor attached to the thyroid cartilage. The sensor is a piezoelectric film (detector size: 10 × 30 mm) that generates an electric charge when bent, and has a wide dynamic range between 0–4 kHz. Respiratory flow was measured using a nasal cannula-type flow sensor (Pro-Tech ProFlow cannula, Sleep Lab Products, USA) and differential pressure transmitter (KL-17; Nagano Keiki, Japan), and was recorded at 1 kHz. Laryngeal motion was recorded at 1 kHz, and the sound signal was recorded at 10 kHz and stored simultaneously with the respiratory signal in a Micro SD card for later analysis. Additionally, we recorded the timing of swallowing for later verification using a foot switch to generate TTL-level pulse signals. The signals were analyzed using MATLAB (R2014b, MathWorks, USA) on a 64-bit Windows 8 professional computer.“

8. Clearly define all swallowing parameters that were quantified - latency, laryngeal motion, respiratory phases etc.

Response: Thank you very much for your very valuable opinion. There was insufficient explanation for each word. According to this comment, we have added details on lines 152-172 as shown below.

“The relationship between swallowing and respiratory cycles was assessed using parameters, including swallowing latency, laryngeal rising time (LRT), laryngeal activation duration (LAD), pause duration, old phase, and co-phase, as previously described [27, 29, 33, 39].

The parameters were defined as follows:

Swallowing latency: The time from the onset of respiratory pause to the onset of the swallowing reflex, defined as the time point when the laryngeal elevation speed reaches its maximum.

LRT: The time required for the larynx to elevate to its highest position.

LAD: The duration between the time of maximal laryngeal elevation speed and return of the larynx to its initial position.

Pause duration: The duration of respiratory pause associated with swallowing.

Old phase: Timing of swallowing in the respiratory cycle, expressed as the onset of the preceding inspiration to the maximum speed of laryngeal elevation (swallowing onset), normalized by the mean length of the respiratory cycle being 1.

Co-phase: The time from the onset of swallowing to immediately following inspiration, normalized by the mean length of the respiratory cycle being 1.

Breathing–swallowing (B-SW) coordination pattern: We classified the breathing–swallowing pattern based on two sets of parameters: (1) B-SW type, which characterizes the interplay between swallowing and the preceding respiratory phase, either E-SW (expiration-swallow) or I-SW (inspiration-swallow); and (2) SW-B type, which characterizes the relationship between swallowing and subsequent respiratory phase, either SW-E (swallow-expiration) or SW-I (swallow-inspiration).

”

9. Describe the food and liquid stimuli used for swallow trials along with preparation protocols.

Response: According to this comment, we have added details on lines 172-175 as shown below.

“Test foods: We utilized soft jelly and water as test foods, ensuring that the soft jelly properties, such as hardness, adhesiveness, and cohesiveness, strictly adhered to the criteria outlined in the Japanese Society of Dysphagia Rehabilitation specification for level 0 dysphagia diet in the International Dysphagia Diet Standardization Initiative report [40]”

10. Elaborate on participant instructions prior to swallow trials and precautions taken.

Response: According to this comment, we have written the following in lines 174 and 179 of the main text.
“Participants were positioned upright in a chair, and they voluntarily swallowed approximately 3 g of level 0 test food from a teaspoon and 3 mL of water from a 5-mL syringe, repeating the process two to five times each. The participants were instructed to swallow the level 0 jelly without chewing. During the examination, the patients were not provided with specific verbal instructions regarding the nature or timing of their swallowing or breathing patterns relative to swallowing.”

11. Explain the statistical tests used for comparing swallowing metrics before and after CRT.
Response: According to this comment, we have described the tests used, including the method for calculating the effect size in lines 182 and 192 of the main text as shown below.
 “To compare variables before and after the CRT, t-tests were performed for swallowing latency, LRT, LAD, pause duration, old phase, and co-phase. Wilcoxon rank-sum tests were performed for the FILS, VE, VF, and EAT10. Comparisons of swallowing patterns (SW-E or SW-I) were performed using χ^2^ or Fisher’s exact tests. The effect size (Cohen’s d) was calculated to assess the substantive impact of CRT on each variable [41]. The categorization of effect sizes, as measured by Cohen’s d, designates a lack of effect when <0.2, a small effect ranging between 0.2–0.5, a moderate effect spanning between 0.5–0.8, and a substantial effect at ≥0.8. The effect size for swallowing patterns was calculated using φ coefficient, which indicates correlation [42]. The categorization of effect sizes, as measured by φ coefficient, designates a lack of effect when <0.1, a small effect ranging between 0.1–0.3, a moderate effect spanning between 0.3–0.5, and a substantial effect at ≥0.5. All statistical analyses were performed using JMP11 software (SAS Institute Inc., Cary, NC, USA). In all instances, a two-sided p-value of <0.05 was considered statistically significant.

”

Results

12. When reporting changes in swallowing metrics before and after CRT, include exact p-values and effect sizes for the comparisons.

Response: According to this comment, effect sizes were calculated and reported in the table and text.

13. For swallowing coordination patterns, provide percentages in addition to p-values when comparing groups.
Response: According to this comment, percentages and P values are listed. Thank you very much for making it very easy to understand. we have written the following in lines 227 and 230 of the main text.

“After the CRT, significantly more SW-I patterns were observed in level 0 food, water, and all conditions than before CRT, with statistical significance of p = 0.0025 (0% vs. 9.2%), p = 0.019 (1.01% vs. 8.57%), and p = 0.0001 (0.52% vs. 8.85%), respectively.”

14. Consider including a table comparing swallowing parameters and coordination patterns before and after CRT.
Response: According to this comment, we combined tables 3 and 4. Visibility has improved, thank you for pointing it out.

15. Mention any differences between liquid and solid swallows in the effects of CRT on breathing-swallowing coordination.
Response: Thank you for your advice. we have listed it in the discussion section.

Discussion

16. Interpret the prolonged swallowing latency and pause duration after CRT in context of known pathophysiological effects of chemoradiation on pharyngeal muscles and sensation.
Response:

According to this comment, we have included the following in the discussion section in lines 248 and 251 as shown below.

“Our results showed significantly prolonged swallowing latency and pause duration after CRT compared with those before CRT. This is due to the previously reported side effects of CRT, such as decreased laryngeal perception, xerostomia, radiation-induced fibrosis of the mucosa associated with the pharyngeal wall, and decreased motility of the oropharynx [9, 10, 15, 16].”

17. Compare the magnitude of increase in SW-I patterns to rates reported for other disorders like stroke, COPD.
Response: According to this comment, we have included the following in lines 255 and 258 as shown below.

“In previous studies monitoring respiratory swallowing, the I-SW and SW-I patterns showed higher frequencies in patients with stroke (18.4%), Parkinson's disease (40.0%), and COPD (13.6%) [44-46]. These rates surpassed those observed in this study.”

18. Discuss possible reasons for SW-I pattern increase specifically after liquid swallows versus solids.

Response: According to this comment, we have included the following in lines 259 and 263 as shown below.
“The prevalence of the SW-I pattern exhibited a notable increase following liquid ingestion in contrast to solid ingestion. This phenomenon could be attributed to differences in the physical attributes of materials, such as viscosity and shape stability, which influence swallowing dynamics, rendering liquids more challenging to swallow than solids [47]. Moreover, it has been suggested that level 0 food, which is deemed the safest for preventing aspiration, is particularly efficacious in risk assessment [34].”

19. Discuss whether SW-I patterns could be a predictive biomarker of aspiration risk in this population.
Response: According to this comment, we have included the following in lines 282 and 287 as shown below.

“Harris et al. demonstrated that a biofeedback approach for coordinating breathing and swallowing improved swallowing function in patients with postoperative patients with head and neck cancer [28]. Early detection and intervention of coordination disorders in breathing and swallowing may be valuable for improving the quality of life of patients. Based on our results, we believe that our methodology, which detects respiratory and swallowing dysfunctions, can serve as a biomarker for the risk of aspiration pneumonia after CRT.

”

20. Suggest future studies to understand neural mechanisms underlying CRT effects on breathing-swallowing coordination.

Response: According to this comment, we have included the following in lines 288 and 295 as shown below.

“To understand the neural mechanisms underlying the effects of CRT on respiratory–swallowing coordination, it is essential to simultaneously measure various parameters. This includes assessing the volume and electromyography of muscles related to swallowing, such as the tongue and pharynx, conducting objective evaluations of pharyngeal perception, and utilizing methods, such as esophageal manometry. This evaluation should be conducted comprehensively and longitudinally.

Moreover, the study participants should encompass a sufficient number of individuals with diverse backgrounds, including not only healthy individuals, but also patients with neurological disorders, COPD, head and neck cancer, and various other conditions.

”
