# Supplementary material for: Breathing–Swallowing discoordination after definitive chemoradiotherapy for head and neck cancers is associated with aspiration pneumonia
Source: PLoS One. 2024 Jul 11;19(7):e0305560. doi: 10.1371/journal.pone.0305560 (PMC11238977; doi:10.1371/journal.pone.0305560)
Supplement: Attachment — Submitted filename: Response to Reviewers .docx [file pone.0305560.s004.docx]

Dear Editors and Reviewers

We sincerely appreciate the editor’s and reviewers’ comments. We revised our article according to the comments.

Thanks to the insightful feedback provided, I am confident that the manuscript has become clearer and more comprehensible. I am deeply grateful for your continued support.

Reviewer #3:

Response: Thank you for your thoughtful review.

Comment:

1.
Methods and Materials
- Swallowing evaluation: I believe elaboration on how swallowing evaluations were conducted would strengthen this manuscript since it the procedures are still unclear.
- [Line 124 - 125] Videoendoscopy and Videofluoroscopy: The methods and results suggest that both a videoendoscopy and videofluoroscopy were completed by participants but it is unclear in what order these assessments were completed. Please clarify the timing of the videoendoscopic and videofluoroscopic evaluations (with respect to each other) and whether swallow monitoring was conducted concurrently.

Response: Thank you for your valuable opinion. According to this comment, we have revised the following.

*All swallowing evaluation were performed on the same day before and one month after CRT.* [Line 125]

2..
- Study Protocols:
- Elaborate on the bolus administration protocols for the videoendoscopic and videofluoroscopic evaluations, especially if they are different from the swallow monitoring bolus protocol.

Response: Thank you for your suggestion, I have made the corrections regarding VE and VF and their respective explanations.

*For the VE evaluation, we used a nasopharyngeal-laryngoscope with a diameter of 3.9 mm with up/down tip deflection capability (Olympus ENF-VH; Olympus Tokyo, Japan) and a digital color video monitor to perform the VE. Patients in the sitting position underwent transnasal endoscopic examinations without nasal anesthetic spray. The Hyodo score was obtained based on endoscopic evaluation of swallowing using 3 ml blue-dyed water with direct visualization of the larynx.* [Line 141-145]

*For the VF evaluation, 3 cm3 of 40% (w/v) barium sulfate (Kaigen Pharma Co., Ltd, Osaka, Japan) with a viscosity of 16 mPa・s was injected into the participant's oral cavity while the evaluator was seated. Then, patients were observed during swallowing to determine whether there was penetration or aspiration, rated by the PAS. The visualization field of fluoroscopic examination extended from the* infraorbital border *to the thoracic esophagus, and evaluations were conducted twice each for anterior and lateral views.* [Line 161-166]

3.
- If barium contrast was used, provide details as to the manufacturer and viscosity.

Response: Thank you for your valuable opinion. According to this comment, we have revised the following.

*For the VF evaluation, 3 cm3 of 40% (w/v) barium sulfate (Kaigen Pharma Co., Ltd, Osaka, Japan) with a viscosity of 16 mPa・s was injected into the participant's oral cavity while the evaluator was seated.* [Line 161]

4.

- Clarify the visualization field and positioning of participant for each of the evaluations.

Response: Thank you for your valuable opinion. According to this comment, we have revised the following.

*Patients in the sitting position underwent transnasal endoscopic examinations without nasal anesthetic spray*. [Line 143-144]

*The visualization field of fluoroscopic examination extended from the* infraorbital border *to the thoracic esophagus, and evaluations were conducted twice each for anterior and lateral views. [*Line 164-166]

5.

For any analyses that were completed, please elaborate on any methods for blinding of studies, raters, inter/intra-rater reliability and any training that the raters may have completed prior to study analysis. -

Response: We appreciate your valuable comments. We have made additions and corrections according to your comments.

*Three experienced Otolaryngology specialists, who have completed swallowing function evaluation training set by the Ministry of Health, Labor, and Welfare and the Society of Swallowing and Dysphagia of Japan, along with a dysphagia-certified nurse and a speech-language pathologist, assessed the swallowing function using VE and VF. All evaluations of VE and VF were recorded in audio-video interleave (AVI) files at a rate of 30 frames per second. All evaluations were discussed on the same day following the examination to achieve inter-rater agreement. VE and VF were performed safely on all patients with no apparent complications*. [Line 134-140]

6.
- [Line 133 - 142] Hyodo score:
- Please specify the equipment used for endoscopic evaluation of swallowing, whether the evaluation was recorded for later review (and any equipment used for recording). If the evaluation was recorded and analyzed, please elaborate on the timing of analysis relative to the endoscopic evaluation (i.e., real-time vs post-hoc), and what software was used for analysis. It may be beneficial to review the FRONTIERS framework sections on Videofluoroscopy or Fiberoptic Endoscopic Evaluation of Swallowing for guidance.

Response: We appreciate your valuable comments. We have made additions and corrections according to your comments.

*All evaluations of VE and VF were recorded in audio-video interleave (AVI) files at a rate of 30 frames per second. All evaluations were discussed on the same day following the examination to achieve inter-rater agreement. VE and VF were performed safely on all patients with no apparent complications*. [Line 138-140]

7.

- Clarify whether lubricant and/or anesthetic was used with any of the participants during the videoendoscopic evaluation.
Response: We appreciate your valuable comments. We have made additions and corrections according to your comments.

*patients in the sitting position underwent transnasal endoscopic examinations without nasal anesthetic spray.* [Line 144]

8.

-- [Line 143 - 149] Penetration-Aspiration Scale Similar to the Hyodo score comments, this section would be strengthened with support of information on how the procedure was conducted and any analysis completed.

Response: Thank you very much for your comment. I have also included the VF evaluation method in the answer to comment 5.

*Three experienced Otolaryngology specialists, who have completed swallowing function evaluation training set by the Ministry of Health, Labor, and Welfare and the Society of Swallowing and Dysphagia of Japan, along with a dysphagia-certified nurse and a speech-language pathologist, assessed the swallowing function using VE and VF. All evaluations of VE and VF were recorded in audio-video interleave (AVI) files at a rate of 30 frames per second. All evaluations were discussed on the same day following the examination to achieve inter-rater agreement. VE and VF were performed safely on all patients with no apparent complications*. [Line 134-145]

9.

Clarify the scoring parameters on the EAT-10 (what 0 vs 4 signify).

Response: We appreciate your valuable comments. We have made additions and corrections according to your comments.

*The score for each item on the EAT10 is 0 indicating no abnormality and 4 indicating severe disability.* [Line 175]

10.

- [Line 161] Swallowing monitoring:
- Elaborate on any synchronization of the swallow evaluation procedures and any processes for aligning events.
- Provide details on the bolus protocol that was planned for participants. Clarify the proportion of participants who were able to complete the full protocol vs those who could not.

Response: Thank you for your valuable feedback. We have added notes on the evaluation procedure, consistency measures, and bolus protocol. We also noted that no participants deviated from the protocol.

*The stored data were subsequently analyzed by a medical engineering research expert and a neurophysiologist specialized in swallowing and respiration. Both analysts were blinded to patient information, and the results were determined through consensus.*

*[Line 191-193]*

*Monitoring of swallowing was performed by two experienced otolaryngology specialists on the same day as the VE and VF evaluations. Before the examination, participants were told that the test would be performed with level 0 food, followed by water, that they would be asked to swallow five times each, that they could stop if they felt pain, that they should not talk during the test, and that they should not chew level 0 food. [Line 217-221]*

*Monitoring of swallowing was performed safely on all patients with no apparent complications. [Line 231]*

11.

Statistical Analyses
- If not all participants could complete the full bolus protocol for any of the swallow evaluations or swallow monitoring, how was missing data handled?
Response: We appreciate your valuable comments. We have added the following information.

*Monitoring of swallowing was performed safely on all patients with no apparent complications. [*Line 231]

12.

Results
- Patient characteristics:
- Table 1: What proportion of participants may have undergone surgical intervention prior to CRT?

Response: We appreciate your valuable comments. We have added the following information.

*All patients received CRT as initial treatment and no prior surgical intervention*. [Line 252]

13.

Discussion
- The majority of the discussion appears focused on respiratory-swallow coordination; the authors should consider discussion of the other measures assessed in this study (i.e., FILS, Hyodo, PAS, EAT-10) in relation to the overall findings.

Response: Thank you very much for your constructive comments. We have revised our discussion as follows.

*Before and after CRT, deterioration in FOIS and EAT10 scores was observed. This suggests that due to CRT, patients experience inhibition of oral intake, indicating the need for some form of nutritional support for the maintenance of vital functions. It also indicates a decrease in quality of life from both functional and psychological perspectives. A patient-completed swallowing screening questionnaire offers a simple and effective tool for daily clinical use, allowing healthcare providers to promptly identify swallowing issues post CRT. This could help in the timely implementation of further assessments such as VF and VE, along with appropriate rehabilitation interventions. In this protocol, the VE evaluation, or Hyodo score, significantly worsened before and after CRT, whereas no significant change was noted in the PAS scale. This suggests that when assessing swallowing after CRT, PAS may not detect changes or may be obscured, particularly with a small bolus condition. While swallowing evaluation with VE can be conducted at the bedside using only an endoscope, VF necessitates relocation to the fluoroscopy room and radiation exposure. In terms of ease of performance, VE and evaluation of breathing-swallowing coordination can be considered advantageous for evaluating swallowing function after CRT.*

*In this study, only small boluses were utilized. The rationale behind this decision was to ensure consistency with the protocols and conditions of prior studies employing noninvasive swallowing measurement system. This approach aimed to investigate variations in the coordination of breathing and swallowing across diverse patient conditions. Increasing bolus size is expected to prolong the time taken for bolus transit relative to smaller boluses, resulting in extended pause duration and potentially exacerbating in breathing-swallowing discoordination. Therefore, the observed breathing-swallowing discoordination in this study may be underestimated compared to evaluations using oral intake or protocols employing larger boluses in clinical practice. Although not included in this protocol, the utilization of various test foods in VE, VF and monitoring of swallowing could allow for the evaluation of swallowing function for each food type. Moreover, by utilizing these assessments to determine the viscosity of safe and suitable foods and rehabilitation strategies based on swallowing function, and subsequently enhancing the level of oral intake, it is conceivable that the quality of life for patients after CRT may be improved.* [Line 366-393]

14.

[Line 314] I believe the following statement, if pertaining to the current study, may require further statistical support that I am unable to identify in the current draft of this manuscript: "In the present study, prolonged swallowing latency after CRT was attributed to edema, fibrous scarring, muscle weakness, and decreased laryngeal sensation due to chemoradiation treatment." If this statement is associated with this manuscript, detailed information regarding the relationship between edema, presence of fibrosis, muscle weakness, and laryngeal sensation should be provided.

Response: Thank you very much for your constructive comments. We have revised our discussion as follows.

*This could potentially be attributed to reported adverse effects of CRT, such as decreased laryngeal perception, xerostomia, radiation-induced fibrosis of the mucosa associated with the pharyngeal wall, and decreased motility of the oropharynx [9, 10, 15, 16]. However, the exact mechanism was not elucidated in this study. Investigating the relationship between swallowing-related muscle strength, motility, laryngeal sensation, saliva volume, and breathing-swallowing coordination through simultaneous measurements would be a prospective focus for future research.* [Line 312-317]

*Although not included in this protocol, the utilization of various test foods in VE, VF and monitoring of swallowing could allow for the evaluation of swallowing function for each food type. Moreover, by utilizing these assessments to determine the viscosity of safe and suitable foods and rehabilitation strategies based on swallowing function, and subsequently enhancing the level of oral intake, it is conceivable that the quality of life for patients after CRT may be improved.* [Line 386-390]

15.
- [Line 323] The authors may wish to verify Dr. Martin-Harris's name within the discussion section
Response: Thank you for pointing this out. We have corrected it correctly.

16.

In the present study, the reported bolus protocol (line 201) is limited to two bolus viscosities of approximately the same size. What influence do the authors think having a more expansive bolus protocol may have on their findings? How does this protocol contrast to other published protocols in terms of external validity?

Response: Thank you for your constructive feedback. according to this comment, comments received, we have added the reasons that led us to this protocol, the possibility that the results may be underestimated, and the expected changes when the bolus is increased.

*In this study, only small boluses were utilized. The rationale behind this decision was to ensure consistency with the protocols and conditions of prior studies employing noninvasive swallowing measurement system. This approach aimed to investigate variations in the coordination of breathing and swallowing across diverse patient conditions. Increasing bolus size is expected to prolong the time taken for bolus transit relative to smaller boluses, resulting in extended pause duration and potentially exacerbating in breathing-swallowing discoordination. Therefore, the observed breathing-swallowing discoordination in this study may be underestimated compared to evaluations using oral intake or protocols employing larger boluses in clinical practice.　 [Line 379-390]*
